# The T-box transcription factor Midline regulates wing development by repressing *wingless* and *hedgehog* in *Drosophila*

**DOI:** 10.1038/srep27981

**Published:** 2016-06-15

**Authors:** Chong-Lei Fu, Xian-Feng Wang, Qian Cheng, Dan Wang, Susumu Hirose, Qing-Xin Liu

**Affiliations:** 1 Laboratory of Developmental Genetics, Shandong Agricultural University, Tai’an, Shandong 271018, China; 2 Department of Developmental Genetics, National Institute of Genetics, Mishima, Shizuoka 411-8540, Japan

## Abstract

Wingless (Wg) and Hedgehog (Hh) signaling pathways are key players in animal development. However, regulation of the expression of *wg* and *hh* are not well understood. Here, we show that Midline (Mid), an evolutionarily conserved transcription factor, expresses in the wing disc of *Drosophila* and plays a vital role in wing development. Loss or knock down of *mid* in the wing disc induced hyper-expression of *wingless* (*wg*) and yielded cocked and non-flat wings. Over-expression of *mid* in the wing disc markedly repressed the expression of *wg*, *DE-Cadherin (DE-Cad)* and *armadillo (arm)*, and resulted in a small and blistered wing. In addition, a reduction in the dose of *mid* enhanced phenotypes of a gain-of-function mutant of *hedgehog (hh).* We also observed repression of *hh* upon overexpression of *mid* in the wing disc. Taken together, we propose that Mid regulates wing development by repressing *wg* and *hh* in *Drosophila*.

The development of multicellular organisms relies on participation and coordination of several signaling pathways that contribute to regulation of organ growth and pattern. Aberrant activity of these pathways can lead to many human diseases including cancers^1^,^2^,^3^,^4^.

The evolutionarily conserved Wg signaling pathway plays a crucial role in growth and pattern formation during development^5^,^6^,^7^,^8^. Signaling molecules of the Wg family are involed in a variety of cellular processes during development and mutations in components of Wg pathway are associated with many cancers^1^,^9^. In the *Drosophila* wing disc, as a gradient morphogen, Wg protein is secreted from the producing cells and acts at long range to activate target gene expression in surrounding cells^10^. Notch (N) induced the expression of *wg* in cells along the dorsal-ventral (DV) boundary^11^,^12^. The Wg/Wnt ligand sends a signal through binding to its receptor Frizzled (Fz) and co-receptor Arrow^13^,^14^. Then a multiprotein complex blocks degradation of Arm, a central component of the Wg pathway. As a consequence, free Arm enters into the nucleus and interacts with TCF/Lef1 to activate transcription of corresponding target genes in response to Wg signaling^15^,^16^,^17^,^18^. The expression of *wg* is activated by Dachs and repressed by Expanded and Hinge1^19^,^20^,^21^.

The Hh signaling pathway is evolutionarily conserved and plays an essential role in embryonic development and adult tissue patterning^22^,^23^,^24^. Deregulation of Hh signaling leads to various human diseases, including many cancers^1^,^25^,^26^. In *Drosophila* wing disc, Hh regulates cell proliferation and cell fate specification^27^. In the posterior (P) compartment, Hh is induced by Engrailed (En) and moves across the anterior-posterior (AP) boundary to form a concentration gradient^25^,^28^. When Hh binds to its receptor Patched (Ptc), Smoothened (Smo) is released from the unliganded Ptc-dependent inhibition^29^. Smo then modulates the activity of a transcription factor Ci^30^,^31^. Then, Ci translocates into the nucleus and activates the expression of target genes, such as-*decapentaplegic* (*dpp*), *ptc* and *en*^32^,^33^. The expression of *hh* is activated by Dlp and repressed by Ihog and Hyd^34^,^35^. Elucidation of the mechanism that regulates these pathways is essential to understanding the development of multicellular organisms.

In the nucleus, several common regulators are shared by more than one pathways, such as: Groucho (Gro), functions as a corepressor in the downstream of the N, Wnt, Hh, Dpp and EGFR pathways^36^,^37^; Lines (lin), serves as mediators of the Wg, N and Hh pathways^38^ and slimb that negatively regulates both Arm and Ci^39^. This raises a possibility that still another factor can coordinate the Wg and Hh signaling pathways.

Mid is the *Drosophila* homolog of Tbx20 in vertebrates. As an evolutionarily conserved T-box family transcription factor, Mid plays a vital role in cell fate specification and tissue morphogenesis during a series of developmental processes^40^,^41^,^42^,^43^,^44^,^45^. During heart development, *mid* and its paralog *H15* are directly activated by Tinman (Tin) and required for the formation and specification of cardioblast^46^,^47^,^48^,^49^. In nervous system, Mid governs axon pathfinding through controlling the expression of multiple components of the two guidance systems, including Frazzled, Robo and Slit^50^. During development of imaginal disc, *mid* and *H15* function as selector genes to specify ventral fate in the leg disc^51^, and function within the Notch-Delta signaling pathway to specify SOP cell fate in the eye disc^52^. However, the function of Mid in wing development remains unclear.

In this study, we analyzed the function of Mid in the wing disc and its role during wing development. Our study reveals a novel role for Mid as an important regulator of the Wg and Hh signaling pathways.

## Results

### Mid expresses in the peripodial epithelium and disc proper cells of the wing disc

**Figure 1:**
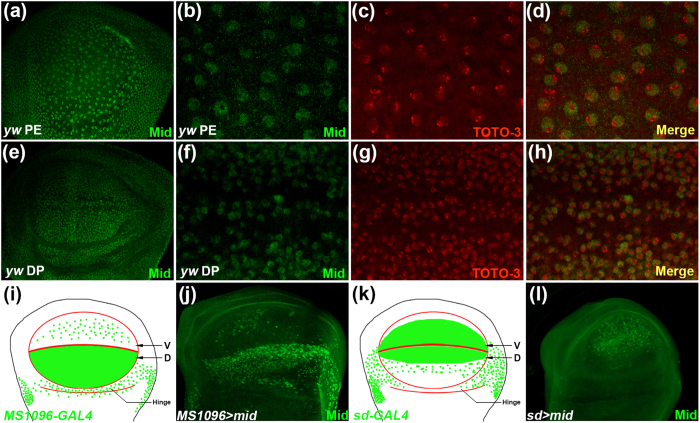
Mid is expressed in the PE and DP of wing discs. (**a–h**) The expression pattern of Mid in the wing disc from a third instar larva of wild type stained with anti-Mid antibody. Optical sections at the plane of the PE (**a–d**) and DP (**e–h**). Mid was detected in the nucleus, labeled by TOTO-3, of both PE (**b–d**) and DP (**f–h**) cells. (**i**) Schematic representation of the *MS1096-GAL4* expression pattern. (**j**) The expression of Mid in the *MS1096-Gal4;UAS-mid* wing disc. (**k**) Schematic representation of the *sd-GAL4* expression pattern. (**l**) The expression of Mid in the *sd-Gal4;UAS-mid* wing disc.

To study the role of Mid in wing development, we first analyzed the expression pattern of Mid in the wing imaginal disc using immunostaining with anti-Mid antibody. In wild-type third instar larvae, Mid ubiquitously expressed in the nucleus marked by TOTO-3 in both peripodial epithelium (PE) (Fig. 1a–d) and the disc proper (DP) cells (Fig. 1e–h). The expression of Mid in the wing disc suggests that it possibly plays a role in wing development.

### Mid plays pivotal roles in wing development

**Figure 2:**
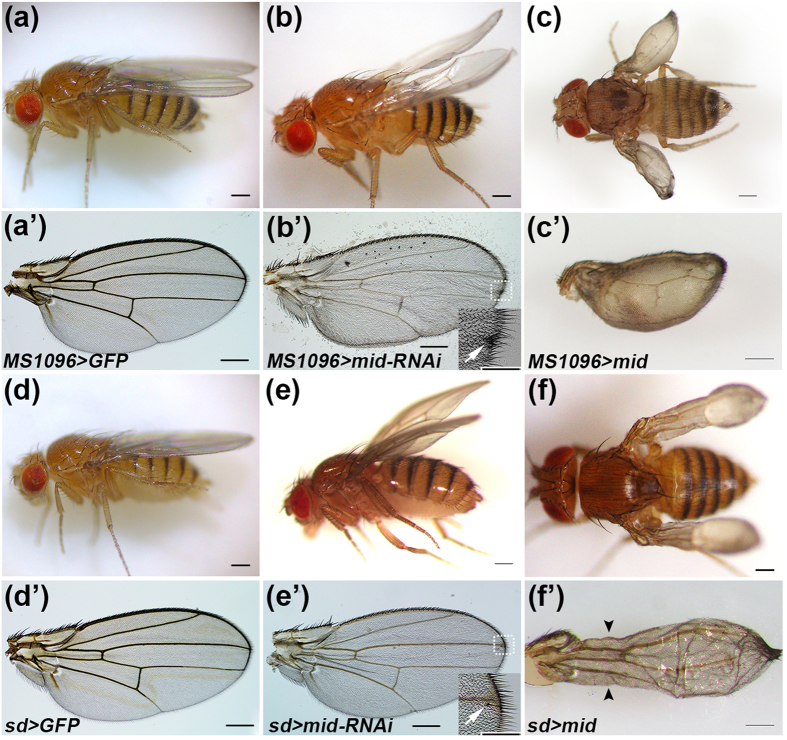
Mid is required for wing development. (**a,a’**) Adult wings of *MS1096-Gal4; UAS-GFP* are shown as control. (**b–c’**) Adult wings of *MS1096-Gal4;UAS-mid-RNAi* (**b,b’**) and *MS1096-Gal4;UAS-mid* (**c,c’).(d,d’**) Adult wings of *sd-Gal4;UAS-GFP* are shown as control. (**e–f’**) Adult wings of *sd-Gal4;UAS-mid-RNAi* (**e,e’**) and *sd-Gal4;UAS-mid* (**f,f’**). Insert of (**b’**) or (**e’**) represents magnification of the region marked by a white broken line. Arrows indicate ectopic wing hairs. Arrowheads indicate loss of tissue at the margin of wing blade. Scale bars: 500 μm.

To gain more insight into the function of Mid in wing development, we analyzed phenotypes observed upon reduction in the *mid* function. Considering the lethality of *mid* null homozygotes during late embryogenesis (*mid*^*1*^ and *mid*^*2*^), we expressed *mid* RNAi using *MS1096*-*GAL4* or *sd*-*GAL4* to knock down *mid* in pouch and margin cells of the wing disc. The expression patterns of *MS1094-GAL4* and *sd-GAL4* are schematically illustrated in Fig. 1(i,k), respectively. Compared with the control wings, knockdown of *mid* resulted in defect wings (Fig. 2a,a’). *MS1096* > *UAS-mid-RNAi* adults exhibited cocked wings with a bowl-shaped depression (Fig. 2b). Likewise, inducing *UAS-mid-RNAi* with *sd-GAL4* resulted in cocked wings with a convex morphology (Fig. 2e). Knockdown of *mid* also induced ectopic wing hairs (Fig. 2b’, Arrows and Figure S1b’, Arrows). When *mid* RNAi was driven by a stronger Gal4 *sd(strong)-GAL4*, we observed a wider space between vein 3 and vein 4 (Figure S1a,b).

To investigate the effect when *mid* was overexpressed in the wing disc, we employed *UAS-mid* transgenic fly. Ectopic expression of *mid* by *MS1096-Gal4* (Fig. 1j) resulted a small and blistered wing with separated surfaces between dorsal and ventral, a phenotype that possibly caused by loss of adhesion between epidermal blades (Fig. 2c,c’). Furthermore, expression of *UAS*-*mid* with *sd*-*GAL4* (Fig. 1l) resulted in loss of tissue at the margin of wing blade, a phenotype typical for a reduction in the Wg signaling (Fig. 2f,f’, Arrowheads). Taken together, loss-of-function and gain-of-function analyses demonstrate that Mid plays a indispensable role in the wing.

### Analysis of candidate downstream target genes of Mid

Given that Mid is a T-box transcription factor^50^, it regulates wing development possibly through activating the expression of target genes. Therefore it will be fruitful to identify the target gene of Mid. To find targets of Mid, we carried out a microarray assay and compared expression profiles of wild-type and *mid* mutant embryos. Among genes that related to wing development, 104 genes were identified as significantly down-regulated and 102 genes were up-regulated in the *mid* mutant (Supplementary Tables S1 and S2). These genes were potential targets of Mid. Among these candidates, we found *wg* and *hh*, which are important for wing development. Given that the phenotypes induced by overexpressing *mid* were analogous to Wg pathway attenuation, and knockdown of *mid* mimicked Hh pathway hyper-activation, we should pay more attention on *hh* and *wg*.

### Mid represses the expression of *wg*

**Figure 3:**
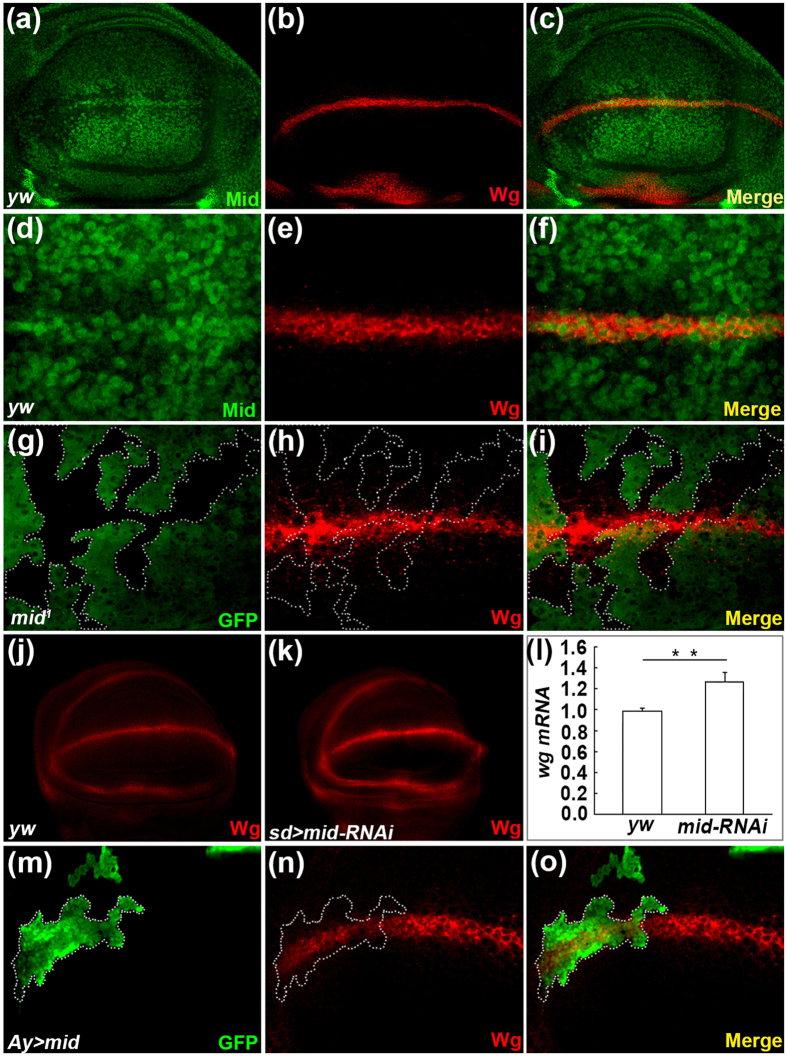
Mid represses the expression of *wg*. (**a–c**) The expression of Mid (**a**) and Wg (**b**) in the wild-type wing disc. (**c**) Merged image of (**a**,**b**). (**d–f**) Magnified images of the partial region in (**a**–**c**) are shown. (**g–i**) Wg expression is activated in a *mid* mutant clone (mutant clone lack GFP signal and marked by a broken line) induced in the DP cells of the wing disc. The wing disc from a third instar larva of *hs-FLP; FRT42D, Ubi-GFP/FRT42D, mid*^*1*^ is stained with anti-GFP antibody (**g**), anti-Wg antibody (**h**). (**i**) Merged image of (**g**,**h**). (**j**,**k**) Activation of the Wg expression in the wing disc of *sd-Gal4; UAS-mid-RNAi* (**k**) compared with that of *yw* (**j**). (**l**) qPCR analyses of the *wg* mRNA level between *yw* and *sd* > *mid-RNAi*. Error bar, SEM from three independent experiments. Student’s tests, **p < 0.01. (**m**–**o**) Somatic clone overexpressing Mid display downregulation of Wg (clone with GFP signal is marked by broken line). The wing disc from a third instar larva of *hs-FLP; UAS-mid/act* > *y* ^+^ > *gal4; UAS-GFP* is stained with anti-GFP antibody (**m**), anti-Wg antibody (**n**). (**o**) Merged image of (**m**,**n**).

To examine a possible role of Mid in the Wg pathway, we first compared the expression patterns of Mid and Wg in the wing disc from third instar larvae. We noticed that Mid and Wg were co-expressed in the DV boundary of DP cells (Fig. 3a–f). The co-localization of Wg and Mid suggests that they have some interaction in the wing disc.

To test whether Mid regulates the *wg* expression, we generated *mid* mutant clones, which effectively depletes the expression of Mid (Figure S2a–c), using FLP-FRT method in the wing disc (Fig. 3g). Compared with the neighboring control cells, *mid* mutant clones marked by loss of GFP showed increased Wg levels (Fig. 3g–i). In addition, we verify this result using RNAi-mediated *mid* knockdown. Knockdown of *mid* with *sd-Gal4* increased the expression of *wg* at the DV boundary (Fig. 3k). Furthermore, we detected the levels of *mid* and *wg* mRNA upon *mid* RNAi by qPCR analyses in the wing disc and found a significantly lower expression level of *mid* mRNA (Figure S3) and a higher expression level of *wg* mRNA (Fig. 3l). These results indicate that *mid* RNAi effectively knocked down *mid* mRNA and that Mid represses the expression of *wg*. We also generated *mid* FLP-out clones in wing disc by the FLP/FRT and the GAL4/UAS techniques. This resulted in reduction of Wg expression (Fig. 3m–o).

To investigate whether cell death increased upon ectopic expression of Mid, we expressed *UAS-mid* with *sd*-*GAL4* and monitored activation of apoptosis by cleaved Caspase-3 antibody. As shown in Figure S4(e), the activity was observed in a small region of the blade but not along the DV boundary, while Wg expression was repressed at the DV boundary (Figure S4d–f). This result suggests that the reduced expression of *wg* upon overexpression of Mid at the DV boundary is not due to increased cell death.

To analyze the genetic interaction between *mid* and *wg*, we induced *wg* RNAi driven by *sd-GAL4* and noted notches at the blade margin (Figure S5b) that are similar to Mid gain of function phenotype (Figure S5a). We then induced *UAS-mid;UAS-wg-RNAi* using *sd-GAL4* and noted a strongly enhanced phenotype, compared with Mid gain of function or Wg knock down alone. Sixty-five percent of the flies exhibit severe loss of wing veins and blistered wing (Figure S5c, n = 99), and 35% of the flies even lead to loss of tissue in almost whole wing blade (Figure S5c’, n = 54). Moreover, an ectopic hair was also found in adults carrying the *mid* mutant clone (Figure S6a’,b’), a phenotype analogous to *wg* ectopic expression.

In sum, our results demonstrate that Mid plays a negative role for *wg* expression in the wing disc.

### Mid negatively regulates the expression of *Arm*

**Figure 4:**
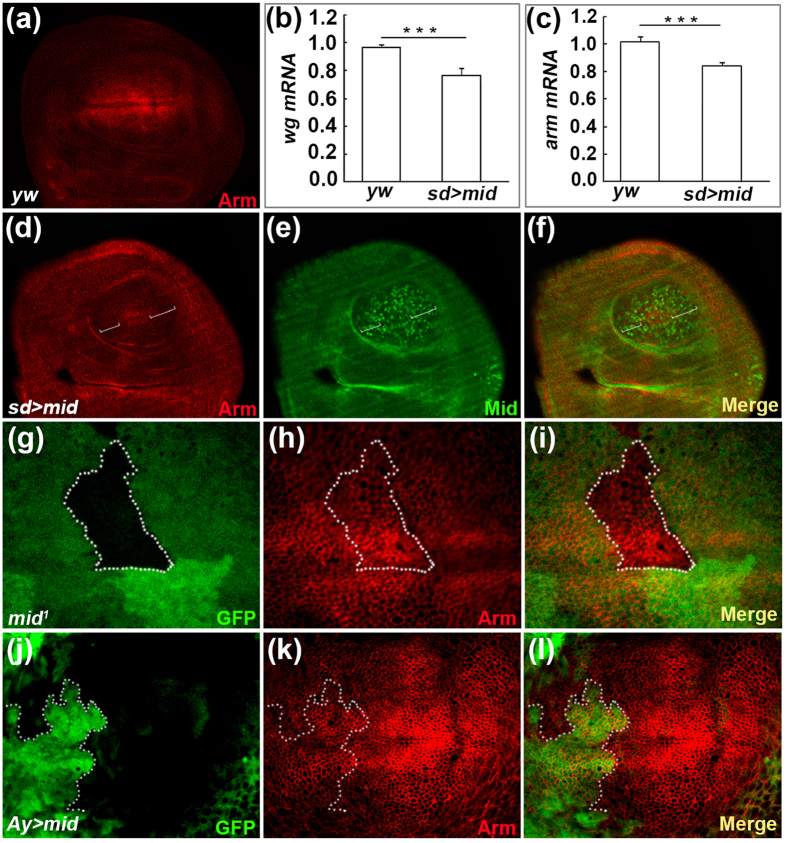
Mid represses the expression of *arm*. (**a**) The wing disc of wild type is stained with the anti-Arm antibody. (**b,c**) qPCR analyses of *wg* and *arm* mRNA level in *yw* and *sd* > *mid*. Error bar, SEM from three independent experiments. Student’s tests, ***p < 0.001. (**d–f**) The expression of Arm is repressed (marked by brackets) in the wing disc of *sd-Gal4; UAS-mid*. (**g–i**) Arm expression is activated in a *mid* mutant clone (mutant clone lack GFP signal and is marked by broken line) induced in the DP cells of the wing disc. The wing disc from a third instar larva of *hs-FLP; FRT42D, Ubi-GFP/FRT42D, mid*^*1*^ is stained with anti-GFP antibody (**g**), anti-Arm antibody (**h**). (**i**) Merged image of (**g**,**h**). (**j**–**l**) Somatic clone overexpressing Mid display downregulation of Arm (clone is marked with GFP signal and is marked by broken line). The wing disc is stained with anti-GFP antibody (**j**), anti-Arm antibody (**k**). (**l**) Merged image of (**j**,**k**).

In order to test whether Mid affects the expression of *wg* downstream genes, we analyzed the expression of Arm/β-catenin. We observed repression of *arm* at the DV boundary when *mid* was overexpressed using the *sd-Gal4* driver (Fig. 4d–f). Furthermore, qPCR analyses revealed that overexpression of *mid* downregulated *wg* and *arm* mRNA levels (Fig. 4b,c). We next generated *mid* loss of function mutant clones (Fig. 4g) and FLP-out clones (Fig. 4j) in the DP cells of the wing disc. Arm expression is increased in *mid* mutant clone in the DV boundary (Fig. 4g–i) and decreased in *mid* overexpression FLP-out clone (Fig. 4j–l). Taken together, these results indicate that Mid negatively regulates the Arm expression in the wing disc.

### Mid negatively regulates the expression of *DE-Cad*

**Figure 5:**
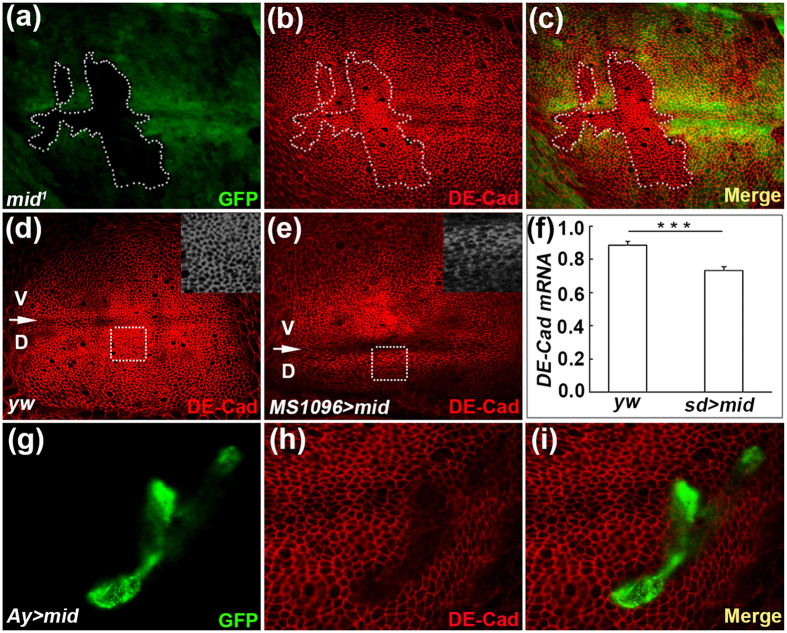
Mid represses the expression of *DE-Cad*. (**a–c**) DE-Cad expression is activated in a *mid* mutant clone (mutant clone lack GFP signal and is marked by a broken line) induced in the DP cells of the wing disc. The wing disc from a third instar larva of *hs-FLP; FRT42D, Ubi-GFP/FRT42D, mid*^*1*^ is stained with anti-GFP antibody (**a**), anti-DE-Cad antibody (**b**). (**c**) Merged image of (**a**,**b**). (**d**,**e**) The expression of DE-Cad in the wing disc of wild type (**d**) and *sd-Gal4; UAS-mid* (**e**). The DV boundary is marked by an arrow. Insert in (**d**) is magnification of the region marked by a white broken line to show normal apical cell shape. Insert in (**e**) is magnification of the region marked by a white broken line to show slender apical cell shape. (**f**) qPCR analyses of *DE-Cad* mRNA level in *yw* and *sd* > *mid*. Error bar, SEM from three independent experiments. Student’s tests, ***p < 0.001. (**g**–**i**) Somatic clone overexpressing Mid display downregulation of DE-Cad (clone is marked with GFP signal). The wing disc is stained with anti-GFP antibody (**g**), anti-DE-Cad antibody (**h**). (**i**) Merged image of (**g**,**h**).

In response to the Wg signaling, DE-Cad regulates epithelial cell-cell adhesion at adherens junctions^53^. To test whether Mid regulates DE-Cad levels, we first induced *mid* loss of function mutant clones in the wing disc, which resulted in hyper-expression of DE-Cad (Fig. 5a–c and Figure S7). We next examined the expression of DE-Cad in wing discs of *yw* and *MS1096-GAL4; UAS-mid*. The control disc displayed a proximodistal (PD) gradient of DE-Cad and normal cell shape (Fig. 5d). However, *mid* overexpression abolished the PD gradient of DE-Cad and resulted in decreased apical cells and cell shape became slender, compared with the same region in the control wing discs (Fig. 5e). In addition, qPCR analyses showed reduced expression of *DE-Cad* when *mid* was overexpressed in wing discs (Fig. 5f). We also overexpressed *mid* in FLP-out clone and found downregulation of DE-Cad (Fig. 5g–i). These results indicate that Mid negatively regulates the expression of *DE-Cad*.

### Mid represses the expression of *hh*

**Figure 6:**
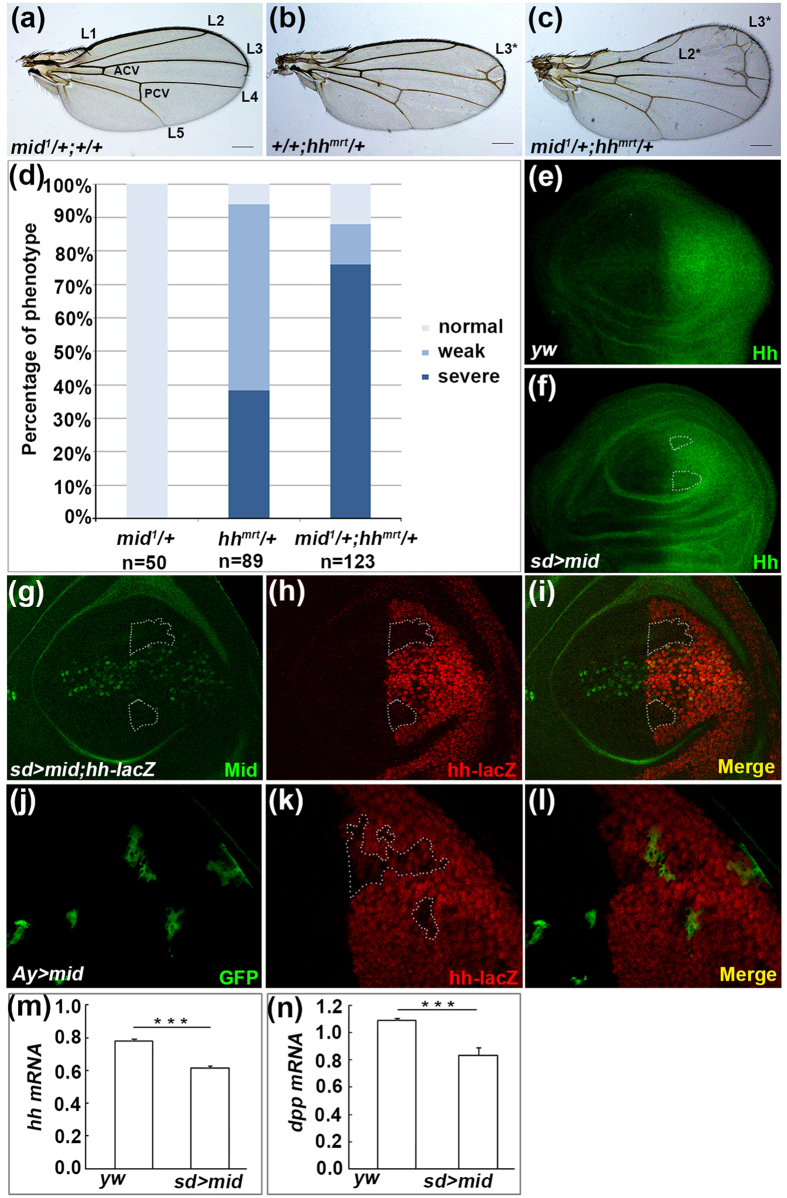
Mid represses the expression of *hh*. (**a–c**) Genetic interaction between *mid* and *hh*. Adult wings of *mid*^*1*^/+; +/+ (**a**) /+; *hh*^*mrt*^/+ (**b**) and *mid*^*1*^/+; *hh*^*mrt*^/+ (**c**). Note ectopic vein L3 (L3*) in (**b**) and ectopic veins L2 (L2*) and L3 (L3*) with enlarged anterior compartment in (**c**). Scale bars: 200 μm. (**d**) Percentage of the phenotype in (**a**–**c**). (**e**,**f**) The wing discs of wild type and *sd* > *mid* are stained with the anti-Hh antibody. The expression of Hh is repressed upon overexpression of *mid* (marked by broken lines in (**f**)). (**g–i**) The wing disc of *sd-Gal4;UAS-mid;hh-lacZ* is stained with the β-gal antibody. The expression of *hh-lacZ* reporter is repressed (marked by broken lines). (**j**–**l**) Somatic clone overexpressing Mid display downregulation of Hh (marked by broken lines). The wing disc from a third instar larva of *hs-FLP; UAS-mid;hh-lacZ/act* > *y* ^+^ > *gal4; UAS-GFP* is stained with anti-GFP antibody (**j**), β-gal antibody (**k**). (**l**) Merged image of (**j**,**k**). (**m,n**) qPCR analyses of the *hh* and *dpp* mRNA levels in *yw* and *sd* > *mid*.

Our microarray data showed enhanced expression of *hh* in the *mid* mutant (Supplementary Table S1), suggesting that Mid is involved in regulation of Hh pathway. To address this possibility, we used *hh* mutant to test whether Hh and Mid have genetic interaction in wings. The *hh*^*Mrt*^ mutant is a gain-of-function allele of *hh*, which leads to ectopic expression of Hh in the anterior compartment and exhibits a wing phenotype with an expanded anterior region and duplication of longitudinal L2 and L3 veins (compare Fig. 6b with 6a)^28^,^54^,^55^. Loss of one copy of *mid* apparently aggravated the phenotypes of *hh*^*Mrt*^ heterozygous adult wing. Compared with the control counterparts, the anterior region of the wing is further enlarged and rounded when *mid* was mutant under *hh*^*Mrt*^ background (Fig. 6c), and ectopic veins L2* and L3* were more obvious. The results were summarized in Fig. 6(d). Moreover, an ectopic vein L3 was also found in adults carrying the *mid* mutant clone (Figure S6a,b), a phenotype analogous to *hh* overexpression^33^. These results suggest that Mid acts as a negative modulator of Hh signaling.

Given that Mid negatively regulates Hh pathway and loss of *mid* increases *hh* mRNA levels, we investigated whether Mid regulates the expression of *hh*. We employed anti-Hh antibody and found that overexpression of *mid* decreased Hh protein levels (Fig. 6e–f). To further confirm that Mid regulates Hh at mRNA level, we used a *hh-lacZ* transgenic fly which could monitor the transcription of *hh*. Overexpression of *mid* repressed *hh-lacZ* expression in a non-cell autonomous manner (Fig. 6g–i). We also examined *hh-lacZ* expression in Mid FLP-out clones and found that reduction of *hh-lacZ* expression still occurs in a non-cell autonomous manner (Fig. 6j–l).

We next examined whether Mid negatively regulates Hh pathway activity. In the wing disc, Hh pathway activates the expression of target genes, including *dpp*. Overexpression of *mid* decreased both *hh* and *dpp* mRNA levels in wing discs (Fig. 6m,n), indicating that Mid represses Hh pathway activity. Taken together, our results demonstrate that Mid inhibits *hh* expression and compromise Hh signaling.

### *mid* is not activated by Wg in the wing disc

Previous studies have revealed that Mid regulates cell fate determination in leg discs, and the expression of *mid* is activated by Wg while repressed by Dpp^51^. It is interesting to examine whether the regulation of Wg upon Mid also occurs in the wing. We cannot analyze adult wings due to lethality caused by *wg* overexpression. Although overexpression of *wg* resulted in a larger wing disc, the level of Mid did not show any detectable changes (Figure S8a–c). We further verified this result using qPCR assays. Overexpression of *wg* apparently increased *wg* mRNA but did not affect *mid* and *hh* mRNA levels (Figure S8d–f). These observations demonstrate that although Mid plays important roles in different tissues, its regulation is distinct. It will be fruitful to investigate how *mid* is regulated in wings.

## Discussion

The Wnt and Hh signaling pathways are involved in many aspects of development and adult homeostasis. Members of the Wg and Hh families induce the expression of a series of target genes by activating conserved signaling cascades^1^,^18^,^56^. Although multiple signaling molecules of the Wg and Hh pathways have been identified, little is known about how these pathways are controlled and coordinated. Here we identified Mid as a common regulator of the Wg and Hh pathways.

**Figure 7:**
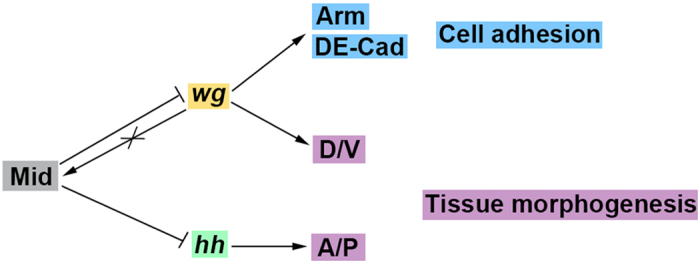
Model for Mid-mediated regulation of wing development. Mid regulates wing development through repression of the Wg and Hh signaling in both tissue morphogenesis and cell adhesion. Mid represses the expression of *wg* to establish a proper expression level of Wg at the DV boundary. The repression of *wg* results in down-regulation of the downstream genes *arm* and *DE-Cad*, which affects the epithelial cell adhesion at adherens junction. By contrast, *wg* does not affect the expression of *mid*. Mid also represses the expression of *hh* to establish a proper expression level of Hh at the P compartment.

We demonstrated that Mid acts as a negative regulator of the Wg pathway (Fig. 7) from three lines of evidence. Firstly, microarray analyses indicated that the level of *wg* mRNA was significantly increased in *mid* mutant compared with the control counterpart. Secondly, loss of *mid* in the wing disc induced hyper-expression of Wg, whereas overexpression of *mid* reduced the expression of Wg. Lastly, Mid acts as a negative regulator of *arm* and *DE-Cad.*

What is the underlying mechanism of the Mid-mediated repression on Wg? Mid could directly repress *wg* together with Groucho as demonstrated in the embryonic ectoderm^41^. However, overexpression of *mid* in the wing disc decreased Wg even in regions where Mid is not overexpressed (our unpublished results), suggesting that Mid represses Wg, at least partly, in an indirect manner. Therefore, Mid possibly attenuates Wg through two mechanisms, direct and indirect. Further studies are necessary to dissect the detailed mechanisms.

We also demonstrated that Mid acts as a negative regulator of the Hh pathway (Fig. 7) from four lines of evidence. Firstly, analyses using microarrays show that the level of *hh* mRNA was enhanced in the *mid* mutant. Secondly, using anti-Hh antibody and *hh-lacZ* reporter, we observed reduced expression of Hh upon overexpression of *mid* in the wing disc. Thirdly, *mid* reduction can enhance the wing phenotype of *hh*^*Mrt*^, a gain-of-function allele of *hh*. Lastly, we observed decrease in the expression of a *hh* target *dpp* in response to overexpression of *mid*. Overexpression of *mid* reduced the expression of *hh-lacZ* in a non-cell autonomous manner. Therefore, the repression of *hh* by Mid is not direct but mediated through an unknown mechanism.

Mid is a homolog of vertebrate Tbx20, an evolutionarily conserved transcription factor that is involved in many important events during development^57^,^58^. In both knock-down and overexpression experiments, we observed abnormalities of wing patterns in response to changes in the level of the *mid* function. Knock down of *mid* in the wing disc induced hyper-expression of Wg and its downstream *DE-Cad*, and yielded the cocked and non-flat wings. The hyper-expression of DE-Cad would lead to abnormally strong cell-cell adhesion and epithelial misfolding^53^. The cocked wing phenotype could be ascribed to the epithelial misfolding in the hinge region. As the *MS1096-gal4* driver is mainly expressed in the dorsal compartment (ref. 59, see also Fig. 1i), *mid* RNAi induced with this driver would lead to hyper-expression of DE-Cad mainly in the dorsal compartment. The resulting strong cell-cell adhesion would reduce the wing surface of the dorsal side. This could give rise to the concave wing. On the other hand, the sd*-gal4* driver mainly expresses in the ventral compartment (ref. 59, see also Fig. 1k) and hence, *mid* RNAi induced with this driver would give rise to the convex wing. Upon knock down of *mid* in the wing disc, we also observed ectopic wing hairs, a phenotype of enhanced *wg* function^39^, and the widening of the distance between L3 and L4 veins, a phenotype of enhanced *hh* function. This is consistent with our conclusion that *mid* regulates wing development by repressing *wg* and *hh*.

Overexpression of *mid* in the wing disc repressed the expression of *wg* and *hh*, which in turn resulted in the small wing phenotype. Overexpression of *mid* in the wing disc also repressed *arm* and *DE-Cad*. Reduced expression of Arm and DE-Cad weakens cell-cell adhesion. Therefore, the blistered wing phenotype upon overexpression of *mid* in the wing disc is most likely due to weakened cell-cell adhesion between the dorsal and the ventral sides.

In sum, we propose a model in which Mid coordinates the Wg and Hh signaling pathways and plays a vital role during wing development (Fig. 7).

## Methods

### Drosophila Stocks

Following stocks were used: *yw*,*mid*^*1*^*/CyO*, *MS1096-GAL4*, *sd(strong)-GAL4*, *hh*^*Mrt*^, *UAS-mid*, *UAS-wg*, *UAS-GFP*, *UAS-wg-RNAi*, *hh-lacZ* were originally obtained from Bloomington Stock Center. *sd-GAL4* was obtained from *Drosophila* Genetic Resource Center. *UAS-mid-RNAi* was originally obtained from NIG-Fly. All fly stocks were grown at 25 °C. Mutant clonal analyses were done with *hs-FLP; FRT42D, Ubi-GFP/CyO* and *FRT42D, mid*^*1*^*/CyO*. FLP-out clonal analyses were done with *hs-FLP; UAS-mid/CyO* and *act* > *y* ^+^ > *gal4;UAS-GFP*.

### Antibodies and Immunohistochemistry

The antibodies were used at the following dilutions: rabbit anti-Mid (1:500^50^), mouse anti-Wg (1:50 DSHB), mouse anti-Arm (1:200 DSHB), rabbit anti-Hh (1:800 gift of T. Tabata), mouse anti-40-1a (1:500 DSHB), 488 donkey anti-rabbit IgG conjugate (1:500, Alexa), Cy3–conjugated donkey anti-mouse IgG (1:500, Sigma), TOTO-3(1:200, Probes) and cleaved Caspase-3(1:200, Cell Signaling).

Images were acquired under Leica TCS SP5 confocal microscope, Olympus DP2-BSW microscope and Olympus cellSens, processed using Adobe Photoshop CS6.

### Microarray Analysis

The *mid* heterozygote or mutant embryos were identified using CyOact-GFP balancer chromosome. Total RNA was extracted separately for each of the heterozygous and homozygous *mid* mutant embryos using the QIAGEN RNeasy isolation kit. The probes labeling and hybridization were performed as described previously^60^. Probes with fold-change >2.0 and P values <0.05 were considered to be differentially expressed. All the Raw and normalized microarray expression data have been deposited on the NCBI Gene Expression Omnibus (GEO) Web site (www.ncbi.nlm.nih.gov/geo) under accession number GSE73802.

### qPCR Analysis

Total RNA was isolated from the wing disc of the third instar larvae using RNAprep Pure Tissue kit (TIANGEN #DP431). Prime Script^TM^ II 1^st^ strand cDNA synthesis kit (TaKaRa #6210A) was used for cDNA synthesis. qPCR was performed in a 20 ul reaction containing 2 pmol of relevant primers (Supplementary Table S3) in Bio-Rad CFX96 real-time system using a SuperReal PreMix Plus (SYBR Green) Kit (TIANGEN #FP205).

## Additional Information

**How to cite this article**: Fu, C.-L. *et al*. The T-box transcription factor Midline regulates wing development by repressing *wingless* and *hedgehog* in *Drosophila*. *Sci. Rep.* **6**, 27981; doi: 10.1038/srep27981 (2016).

## Supplementary Material

Supplementary Information

## References

[b1] Taipale J. & Beachy P. A. The Hedgehog and Wnt signalling pathways in cancer. Nature 411, 349–354, 10.1038/35077219 (2001). 11357142

[b2] Beachy P. A., Karhadkar S. S. & Berman D. M. Tissue repair and stem cell renewal in carcinogenesis. Nature 432, 324–331, 10.1038/nature03100 (2004). 15549094

[b3] Teglund S. & Toftgard R. Hedgehog beyond medulloblastoma and basal cell carcinoma. Biochimica et biophysica acta 1805, 181–208, 10.1016/j.bbcan.2010.01.003 (2010). 20085802

[b4] Weng A. P. . Activating mutations of NOTCH1 in human T cell acute lymphoblastic leukemia. Science (New York, N.Y.) 306, 269–271, 10.1126/science.1102160 (2004). 15472075

[b5] Bejsovec A. & Martinez Arias A. Roles of wingless in patterning the larval epidermis of Drosophila. Development 113, 471–485 (1991). 1782860 10.1242/dev.113.2.471

[b6] Couso J. P., Bishop S. A. & Martinez Arias A. The wingless signalling pathway and the patterning of the wing margin in Drosophila. Development 120, 621–636 (1994). 8162860 10.1242/dev.120.3.621

[b7] Willert K. . Wnt proteins are lipid-modified and can act as stem cell growth factors. Nature 423, 448–452, 10.1038/nature01611 (2003). 12717451

[b8] Hartmann C. A Wnt canon orchestrating osteoblastogenesis. Trends in cell biology 16, 151–158, 10.1016/j.tcb.2006.01.001 (2006). 16466918

[b9] Clevers H. Wnt/beta-catenin signaling in development and disease. Cell 127, 469–480, 10.1016/j.cell.2006.10.018 (2006). 17081971

[b10] Zecca M., Basler K. & Struhl G. Direct and long-range action of a wingless morphogen gradient. Cell 87, 833–844 (1996). 8945511 10.1016/s0092-8674(00)81991-1

[b11] Diaz-Benjumea F. J. & Cohen S. M. Serrate signals through Notch to establish a Wingless-dependent organizer at the dorsal/ventral compartment boundary of the Drosophila wing. Development 121, 4215–4225 (1995). 8575321 10.1242/dev.121.12.4215

[b12] Rulifson E. J., Micchelli C. A., Axelrod J. D., Perrimon N. & Blair S. S. Wingless refines its own expression domain on the Drosophila wing margin. Nature 384, 72–74, 10.1038/384072a0 (1996). 8900280

[b13] Bhanot P. . A new member of the frizzled family from Drosophila functions as a Wingless receptor. Nature 382, 225–230, 10.1038/382225a0 (1996). 8717036

[b14] Cadigan K. M., Fish M. P., Rulifson E. J. & Nusse R. Wingless repression of Drosophila frizzled 2 expression shapes the Wingless morphogen gradient in the wing. Cell 93, 767–777 (1998). 9630221 10.1016/s0092-8674(00)81438-5

[b15] Nelson W. J. & Nusse R. Convergence of Wnt, beta-catenin, and cadherin pathways. Science (New York, N.Y.) 303, 1483–1487, 10.1126/science.1094291 (2004). PMC3372896 15001769

[b16] He X., Saint-Jeannet J. P., Woodgett J. R., Varmus H. E. & Dawid I. B. Glycogen synthase kinase-3 and dorsoventral patterning in Xenopus embryos. Nature 374, 617–622, 10.1038/374617a0 (1995). 7715701

[b17] Daniels D. L., Eklof Spink K. & Weis W. I. Beta-catenin: molecular plasticity and drug design. Trends in biochemical sciences 26, 672–678 (2001). 11701326 10.1016/s0968-0004(01)01952-1

[b18] Logan C. Y. & Nusse R. The Wnt signaling pathway in development and disease. Annual review of cell and developmental biology 20, 781–810, 10.1146/annurev.cellbio.20.010403.113126 (2004). 15473860

[b19] Pellock B. J., Buff E., White K. & Hariharan I. K. The Drosophila tumor suppressors Expanded and Merlin differentially regulate cell cycle exit, apoptosis, and Wingless signaling. Developmental biology 304, 102–115, 10.1016/j.ydbio.2006.12.021 (2007). 17258190 PMC1924969

[b20] Cho E. & Irvine K. D. Action of fat, four-jointed, dachsous and dachs in distal-to-proximal wing signaling. Development 131, 4489–4500, 10.1242/dev.01315 (2004). 15342474

[b21] Shukla V., Habib F., Kulkarni A. & Ratnaparkhi G. S. Gene duplication, lineage-specific expansion, and subfunctionalization in the MADF-BESS family patterns the Drosophila wing hinge. Genetics 196, 481–496, 10.1534/genetics.113.160531 (2014). 24336749 PMC3914621

[b22] Briscoe J. & Therond P. P. The mechanisms of Hedgehog signalling and its roles in development and disease. Nature reviews. Molecular cell biology 14, 416–429, 10.1038/nrm3598 (2013). 23719536

[b23] Ingham P. W. & McMahon A. P. Hedgehog signaling in animal development: paradigms and principles. Genes & development 15, 3059–3087, 10.1101/gad.938601 (2001). 11731473

[b24] Jia J. & Jiang J. Decoding the Hedgehog signal in animal development. Cellular and molecular life sciences: CMLS 63, 1249–1265, 10.1007/s00018-005-5519-z (2006). 16596340 PMC11136406

[b25] Jiang J. & Hui C. C. Hedgehog signaling in development and cancer. Developmental cell 15, 801–812, 10.1016/j.devcel.2008.11.010 (2008). 19081070 PMC6443374

[b26] Pasca di Magliano M. & Hebrok M. Hedgehog signalling in cancer formation and maintenance. Nature reviews. Cancer 3, 903–911, 10.1038/nrc1229 (2003). 14737121

[b27] Bischoff M. . Cytonemes are required for the establishment of a normal Hedgehog morphogen gradient in Drosophila epithelia. Nature cell biology 15, 1269–1281, 10.1038/ncb2856 (2013). 24121526 PMC3840581

[b28] Bejarano F., Perez L., Apidianakis Y., Delidakis C. & Milan M. Hedgehog restricts its expression domain in the Drosophila wing. EMBO reports 8, 778–783, 10.1038/sj.embor.7401003 (2007). 17571073 PMC1978085

[b29] Marigo V., Davey R. A., Zuo Y., Cunningham J. M. & Tabin C. J. Biochemical evidence that patched is the Hedgehog receptor. Nature 384, 176–179, 10.1038/384176a0 (1996). 8906794

[b30] Michelson A. M. Running interference for hedgehog signaling. Science’s STKE: signal transduction knowledge environment 2003, Pe30, 10.1126/stke.2003.192.pe30 (2003). 12881613

[b31] Zhao Y., Tong C. & Jiang J. Hedgehog regulates smoothened activity by inducing a conformational switch. Nature 450, 252–258, 10.1038/nature06225 (2007). 17960137

[b32] Pan D. & Rubin G. M. cAMP-dependent protein kinase and hedgehog act antagonistically in regulating decapentaplegic transcription in Drosophila imaginal discs. Cell 80, 543–552 (1995). 7867062 10.1016/0092-8674(95)90508-1

[b33] Methot N. & Basler K. An absolute requirement for Cubitus interruptus in Hedgehog signaling. Development 128, 733–742 (2001). 11171398 10.1242/dev.128.5.733

[b34] Yan D. . The cell-surface proteins Dally-like and Ihog differentially regulate Hedgehog signaling strength and range during development. Development 137, 2033–2044, 10.1242/dev.045740 (2010). 20501592 PMC2875843

[b35] Lee J. D., Amanai K., Shearn A. & Treisman J. E. The ubiquitin ligase Hyperplastic discs negatively regulates hedgehog and decapentaplegic expression by independent mechanisms. Development 129, 5697–5706 (2002). 12421709 10.1242/dev.00159

[b36] Buscarlet M. & Stifani S. The ‘Marx’ of Groucho on development and disease. Trends in cell biology 17, 353–361, 10.1016/j.tcb.2007.07.002 (2007). 17643306

[b37] Hasson P. . EGFR signaling attenuates Groucho-dependent repression to antagonize Notch transcriptional output. Nature genetics 37, 101–105, 10.1038/ng1486 (2005). 15592470

[b38] Benitez E., Bray S. J., Rodriguez I. & Guerrero I. Lines is required for normal operation of Wingless, Hedgehog and Notch pathways during wing development. Development 136, 1211–1221, 10.1242/dev.021428 (2009). 19270177 PMC2685938

[b39] Jiang J. & Struhl G. Regulation of the Hedgehog and Wingless signalling pathways by the F-box/WD40-repeat protein Slimb. Nature 391, 493–496, 10.1038/35154 (1998). 9461217

[b40] Buescher M. . Functions of the segment polarity genes midline and H15 in Drosophila melanogaster neurogenesis. Developmental biology 292, 418–429, 10.1016/j.ydbio.2006.01.016 (2006). 16499900

[b41] Formaz-Preston A., Ryu J. R., Svendsen P. C. & Brook W. J. The Tbx20 homolog Midline represses wingless in conjunction with Groucho during the maintenance of segment polarity. Developmental biology 369, 319–329, 10.1016/j.ydbio.2012.07.004 (2012). 22814213

[b42] Manavalan M. A., Gaziova I. & Bhat K. M. The Midline Protein Regulates Axon Guidance by Blocking the Reiteration of Neuroblast Rows within the Drosophila Ventral Nerve Cord. PLoS genetics 9, e1004050, 10.1371/journal.pgen.1004050 (2013). 24385932 PMC3873230

[b43] Chen Q. B. . The drosophila T-box transcription factor midline functions within Insulin/Akt and c-Jun-N terminal kinase stress-reactive signaling pathways to regulate interommatial bristle formation and cell survival. Mechanisms of development 136, 8–29, 10.1016/j.mod.2015.02.005 (2015). 25748605 PMC4479208

[b44] Kumar R. P., Dobi K. C., Baylies M. K. & Abmayr S. M. Muscle cell fate choice requires the T-box transcription factor midline in Drosophila. Genetics 199, 777–791, 10.1534/genetics.115.174300 (2015). 25614583 PMC4349071

[b45] Buescher M. . Drosophila T box proteins break the symmetry of hedgehog-dependent activation of wingless. Current biology: CB 14, 1694–1702, 10.1016/j.cub.2004.09.048 (2004). 15458640

[b46] Ryu J. R., Najand N. & Brook W. J. Tinman is a direct activator of midline in the Drosophila dorsal vessel. Developmental dynamics: an official publication of the American Association of Anatomists 240, 86–95, 10.1002/dvdy.22495 (2011). 21108319

[b47] Miskolczi-McCallum C. M., Scavetta R. J., Svendsen P. C., Soanes K. H. & Brook W. J. The Drosophila melanogaster T-box genes midline and H15 are conserved regulators of heart development. Developmental biology 278, 459–472, 10.1016/j.ydbio.2004.11.026 (2005). 15680363

[b48] Qian L., Liu J. & Bodmer R. Neuromancer Tbx20-related genes (H15/midline) promote cell fate specification and morphogenesis of the Drosophila heart. Developmental biology 279, 509–524, 10.1016/j.ydbio.2005.01.013 (2005). 15733676

[b49] Reim I., Mohler J. P. & Frasch M. Tbx20-related genes, mid and H15, are required for tinman expression, proper patterning, and normal differentiation of cardioblasts in Drosophila. Mechanisms of development 122, 1056–1069, 10.1016/j.mod.2005.04.006 (2005). 15922573

[b50] Liu Q. X. . Midline governs axon pathfinding by coordinating expression of two major guidance systems. Genes & development 23, 1165–1170, 10.1101/gad.1774209 (2009). 19451216 PMC2685537

[b51] Svendsen P. C., Formaz-Preston A., Leal S. M. & Brook W. J. The Tbx20 homologs midline and H15 specify ventral fate in the Drosophila melanogaster leg. Development 136, 2689–2693, 10.1242/dev.037911 (2009). 19605497

[b52] Das S. . The Drosophila T-box transcription factor Midline functions within the Notch-Delta signaling pathway to specify sensory organ precursor cell fates and regulates cell survival within the eye imaginal disc. Mechanisms of development 130, 577–601, 10.1016/j.mod.2013.08.001 (2013). 23962751 PMC4500660

[b53] Jaiswal M., Agrawal N. & Sinha P. Fat and Wingless signaling oppositely regulate epithelial cell-cell adhesion and distal wing development in Drosophila. Development 133, 925–935, 10.1242/dev.02243 (2006). 16452097

[b54] Tabata T. & Kornberg T. B. Hedgehog is a signaling protein with a key role in patterning Drosophila imaginal discs. Cell 76, 89–102 (1994). 8287482 10.1016/0092-8674(94)90175-9

[b55] Felsenfeld A. L. & Kennison J. A. Positional signaling by hedgehog in Drosophila imaginal disc development. Development 121, 1–10 (1995). 7867491 10.1242/dev.121.1.1

[b56] Mosimann C., Hausmann G. & Basler K. Parafibromin/Hyrax activates Wnt/Wg target gene transcription by direct association with beta-catenin/Armadillo. Cell 125, 327–341, 10.1016/j.cell.2006.01.053 (2006). 16630820

[b57] Gibson-Brown J. J. . Evidence of a role for T-box genes in the evolution of limb morphogenesis and the specification of forelimb/hindlimb identity. Mechanisms of development 56, 93–101 (1996). 8798150 10.1016/0925-4773(96)00514-x

[b58] Logan M. & Tabin C. J. Role of Pitx1 upstream of Tbx4 in specification of hindlimb identity. Science (New York, N.Y.) 283, 1736–1739 (1999). 10.1126/science.283.5408.1736 10073939

[b59] Bejarano F. . A genome-wide transgenic resource for conditional expression of Drosophila microRNAs. Development 139, 2821–2831, 10.1242/dev.079939 (2012). 22745315 PMC3392707

[b60] Liu Q. X. . Evolutionarily conserved transcription factor Apontic controls the G1/S progression by inducing cyclin E during eye development. Proceedings of the National Academy of Sciences of the United States of America 111, 9497–9502, 10.1073/pnas.1407145111 (2014). 24979795 PMC4084451

