# Supplementary material for: The T-box transcription factor Midline regulates wing development by repressing wingless and hedgehog in Drosophila
Source: Sci Rep. 2016 Jun 15;6:27981. doi: 10.1038/srep27981 (PMC4908378; doi:10.1038/srep27981)
Supplement: Supplementary Information [file srep27981-s1.pdf]

Supplementary information

The T-box transcription factor Midline regulates wing development by repressing *wingless* and *hedgehog* in *Drosophila*

Chong-Lei Fu[1], Xian-Feng Wang[1], Qian Cheng[1], Dan Wang[1], Susumu Hirose[2] and Qing-Xin Liu[1*]

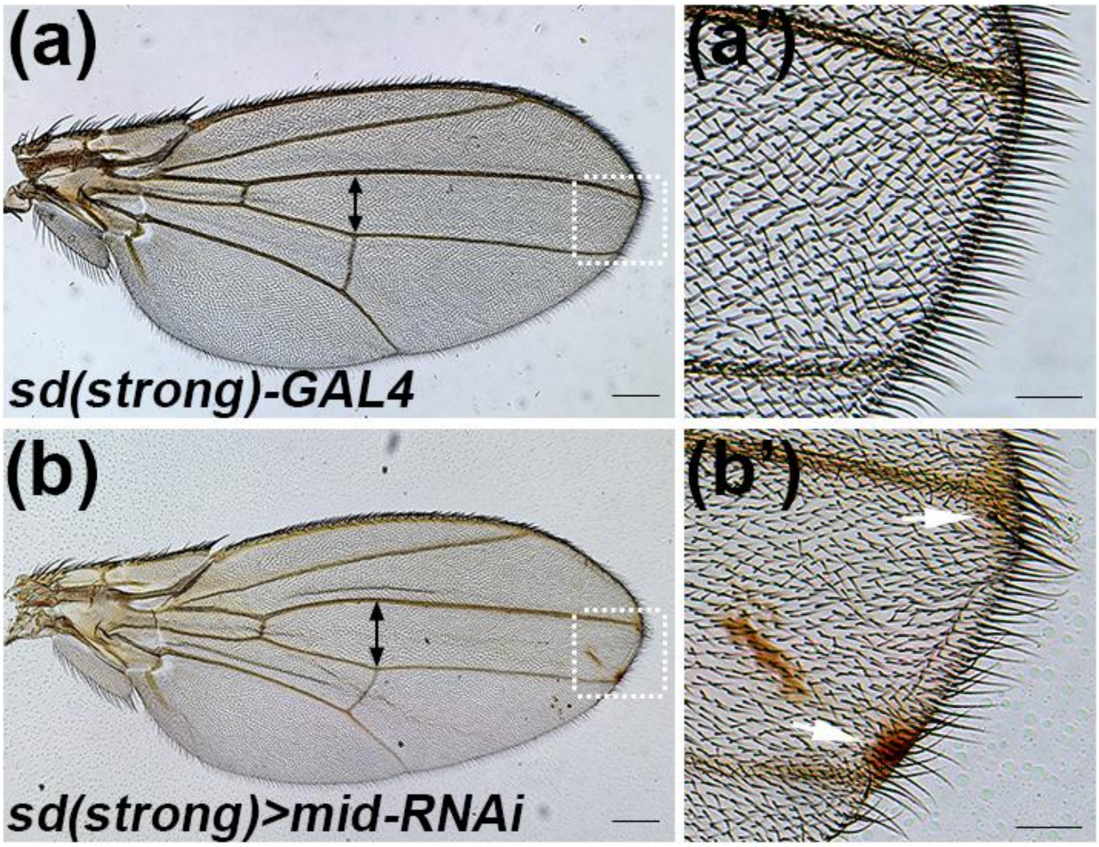

Figure S1. *mid* RNAi induces abnormal wing hairs and vein.

(**a, a'**) Adult wing of *sd(strong)-GAL4.* (**b, b'**) Adult wing of *sd(strong)-GAL4; UAS-mid-RNAi*. (**a', b'**) Magnification of the region marked by a white broken line. Double arrows indicate the distance between L3 and L4. Arrows indicate ectopic wing hairs.

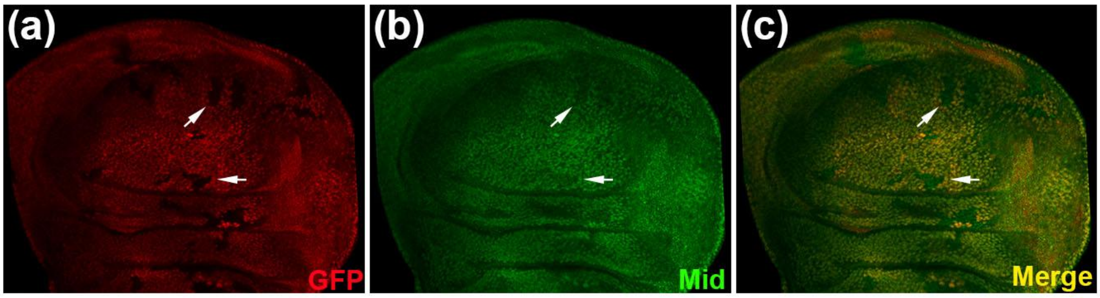

Figure S2. Knockdown of *mid* in mutant clone effectively depletes the expression of Mid.

(**a-c**) Mid expression is effectively depleted (indicated by arrows) in *mid* mutant clones (mutant clone lack GFP signal) induced in the DP cells of the wing disc. The wing disc from a third instar larva of *hs-FLP; FRT42D, Ubi-GFP/FRT42D, mid*^{1} is stained with anti-GFP antibody (**a**), anti-Mid antibody (**b**). (**c**) Merged image of (**a**) and (**b**).

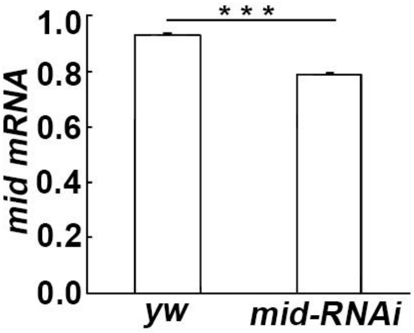

Figure S3. *mid-RNAi* effectively knocked down *mid* mRNA.

qPCR analyses of the *mid* mRNA levels in *yw* and *sd>mid-RNAi*. Error bar, SEM from three independent experiments. Student's tests, ***p<0.001.

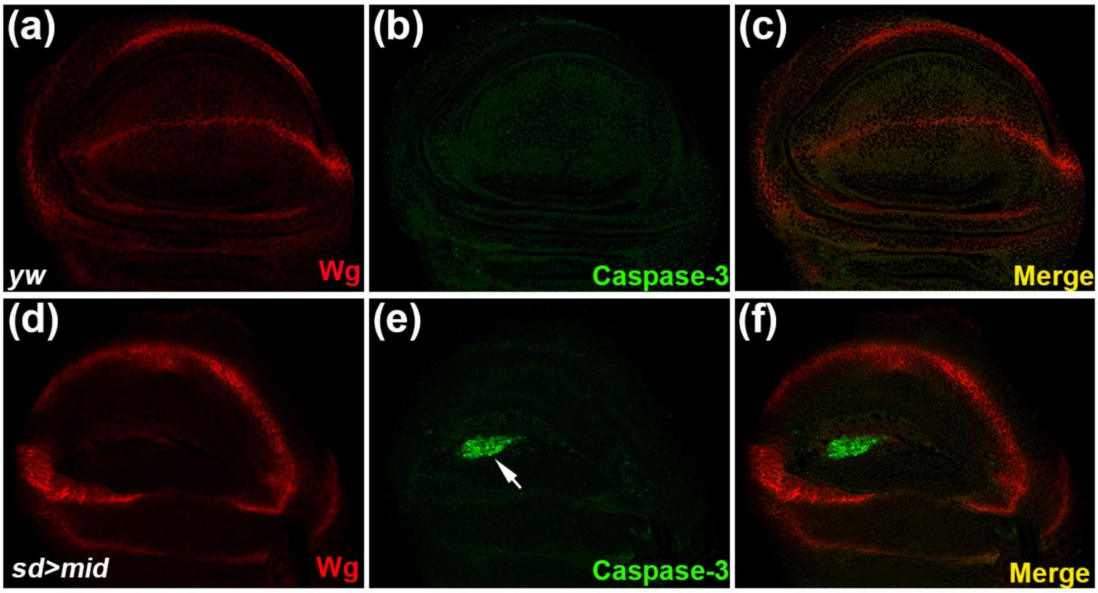

Figure S4. Overexpression of Mid represses *wg* at the DV boundary and induces apoptosis in the wing disc.

(**a-c**) The wing disc of *yw* is stained with the anti-Wg and cleaved Caspase-3 antibody. (**d-f**) The expression of Wg is repressed in the wing disc of *sd-Gal4; UAS-mid* (**d**). Activation of apoptosis (indicated by an arrow) is probed with cleaved Caspase-3 antibody (**e**). (**f**) Merged image of (**d**) and (**e**).

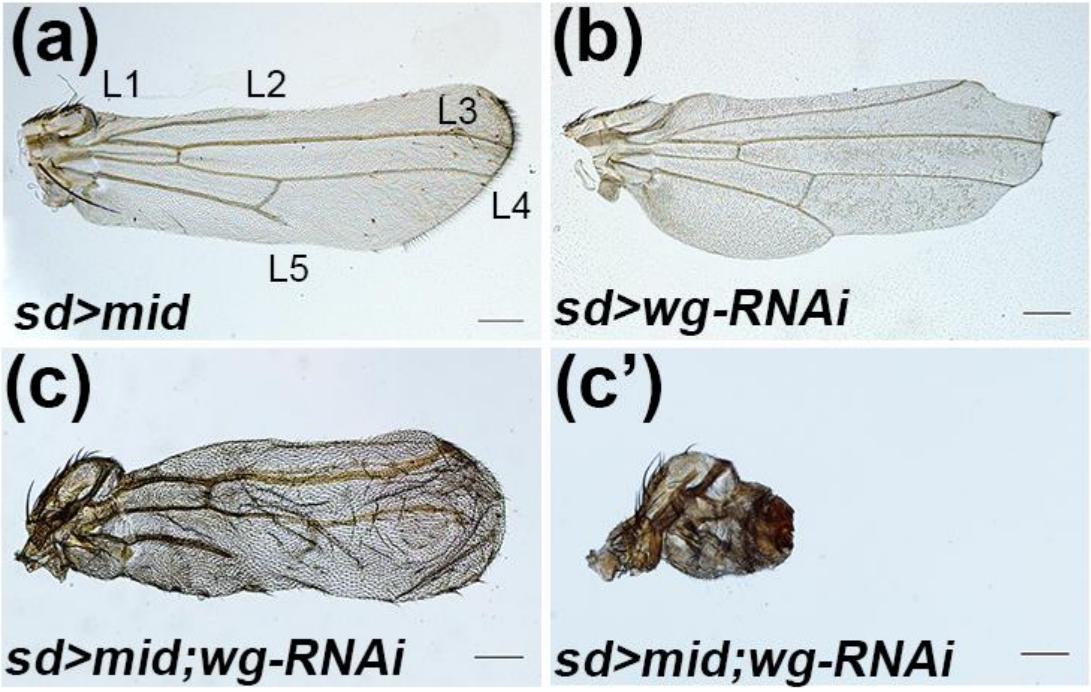

Figure S5. Genetic interaction between *mid* and *wg*.

(**a-c'**) Adult wings of *sd-Gal4; UAS-mid* (**a**), *sd-Gal4; UAS-wg-RNAi* (**b**) and *sd-Gal4; UAS-mid; UAS-wg-RNAi* which exhibits severe loss of wing veins, blistered wing (**c**) and even leads to loss of tissue in almost whole wing blade (**c'**). Scale bars: 200μm.

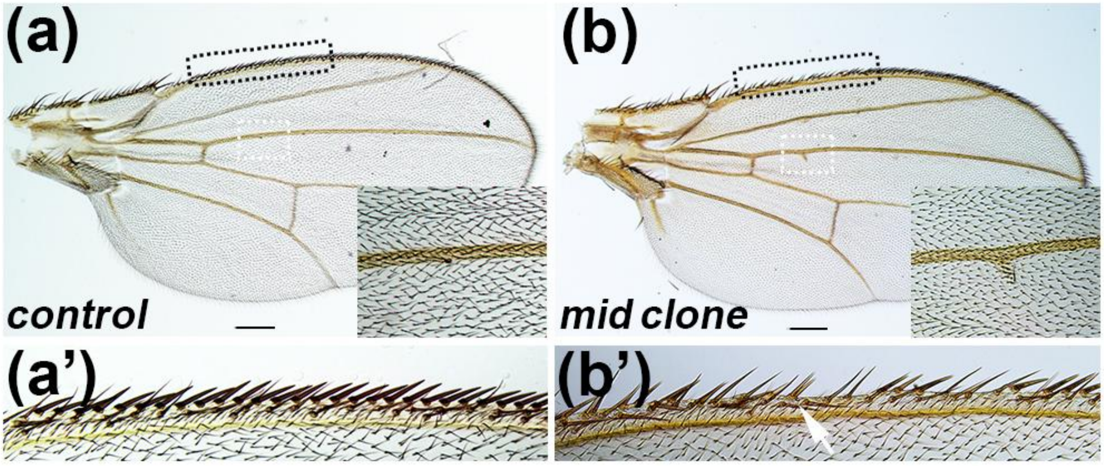

Figure S6. *mid* mutant clone induces an ectopic vein and hair at the anterior compartment.

(**a-b'**) Adult wings of control (**a, a'**) and *mid* mutant clone (**b, b'**). Insert in (**a**) is magnification of the region marked by a white broken line to show normal vein. Insert in (**b**) is magnification of the region marked by a white broken line to show an ectopic vein L3. (**a', b'**) Magnification of the region marked by a black broken line. Arrows indicate ectopic wing hairs.

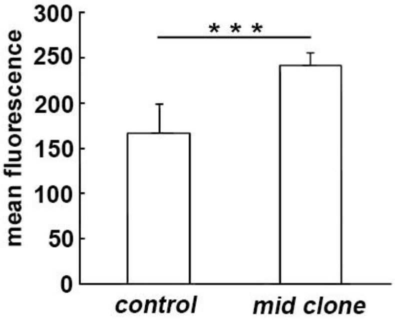

Figure S7. The expression of DE-Cad was induced in *mid* mutant clone.

The expression levels of DE-Cad were determined by mean fluorescence both inside and outside (control) of *mid* clone. Error bars, SEM. ***p < 0.001.

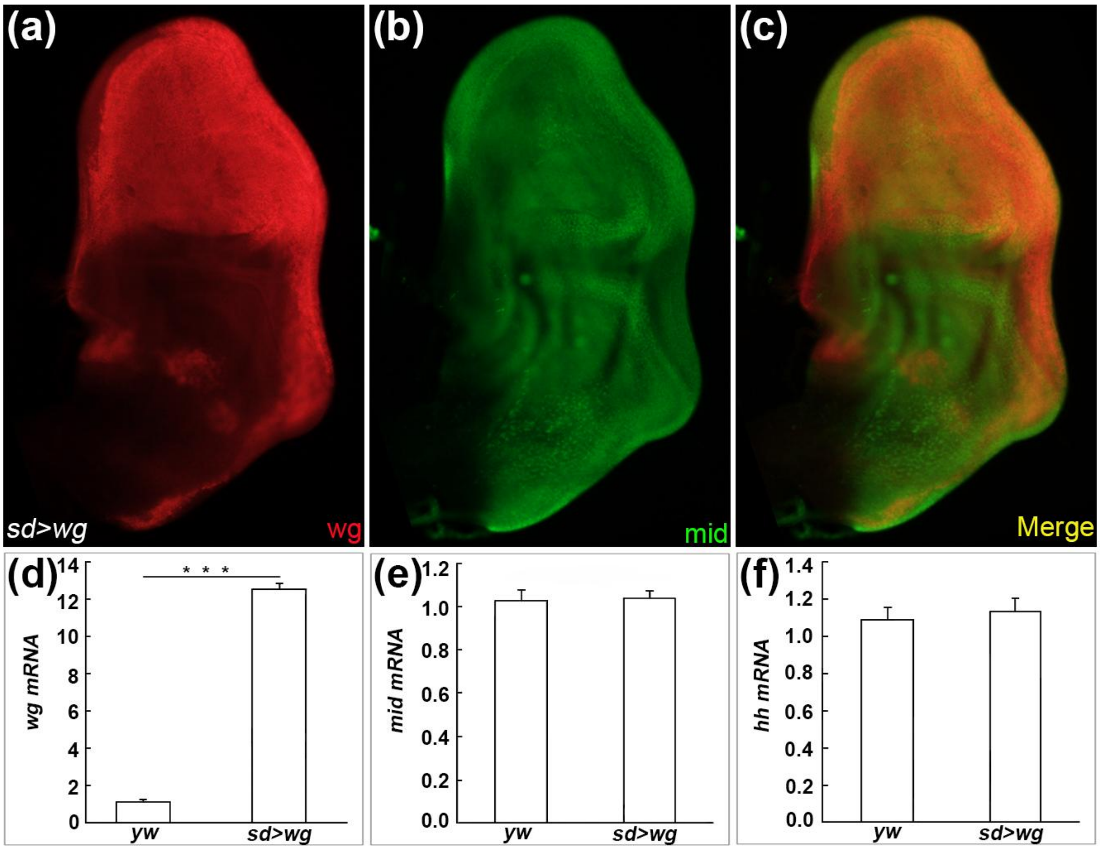

Figure S8. Wg does not activate *mid* in the wing disc.

(**a-c**) The expression of Wg and Mid in *sd-Gal4; UAS-wg*. (**d-f**) qPCR analyses of the *wg*, *mid* and *hh* mRNA levels in *yw* and *sd>wg*. Error bar, SEM from three independent experiments. Student's tests, ***$p<0.001$.

Gene symbol					
CG16884	*wg*	*Wnt4*	*CG11334*	*fz*	*Sxl*
cad	*mirr*	*tld*	*Burs*	*pan*	*fz2*
wupA	*ast*	*dm*	*unk*	*stck*	*rn*
ab	*Btk29A*	*wapl*	*ubx*	*CG3167*	*N*
Irk2	*InR*	*esg*	*argos*	*shot*	*CG16801*
rho	*fng*	*toc*	*wdn*	*kuz*	*os*
Ddc	*hdc*	*emc*	*nmo*	*dpp*	*ppl*
Antp	*babo*	*sgg*	*CG15154*	*ss*	*pyd*
CG18144	*shg*	*Bx*	*dve*	*hh*	*ewg*
apt	*dlg1*	*Scr1*	*ctp*	*CtBP*	*Su(H)*
sn	*neur*	*spi*	*Chi*	*dsh*	*arm*
hth	*CG10369*	*wb*	*sax*	*ara*	*Pka-C1*
en	*LanA*	*bun*	*Dl*	*smo*	*phl*
sgl	*Dad*	*shn*	*sema-1b*	*pnt*	*CG1848*
kn	*trn*	*CG2446*	*Rnk*	*aay*	*Smox*
how	*vg*	*flw*	*ci*	*if*	*ac*
CG6831	*cora*	*Set2*	*caps*	*Ssdp*	*fj*

Table S1. Up-regulated genes in *mid* mutant among wing development-related genes.

Gene symbol					
Cdc42	*Rho1*	*CG6890*	*salm*	*Kr*	*fru*
Fpps	*mys*	*put*	*eIF-5A*	*baz*	*sca*
Klp64D	*Skp2*	*ap*	*tsr*	*Egfr*	*dx*
sog	*S*	*Ser*	*Hydr2*	*CG9491*	*gbb*
Asx	*sd*	*CG3143*	*mew*	*ped*	*svr*
cos	*aret*	*Med*	*brk*	*su(Hw)*	*sqh*
bcn92	*CG5446*	*ato*	*Dll*	*awd*	*mam*
spen	*lola*	*dystrophin*	*Ark*	*CG1518*	*EcR*
CycE	*CG11893*	*malpha*	*nub*	*CG17090*	*CG2767*
gro	*H*	*fog*	*Sb*	*cpb*	*CH9932*
CG8201	*wts*	*elB*	*Hrs*	*scb*	*zip*
E2F	*hop*	*Sin3A*	*CG2604*	*Crc*	*CG1070*
ck	*CG2245*	*CG9867*	*Rbf*	*CG4080*	*CG3564*
dwg	*fw*	*inx7*	*dsx*	*CG4451*	*vri*
sl	*CG8222*	*stan*	*CG12163*	*CG14204*	*bib*
drk	*Elongin-B*	*Tango14*	*Psn*	*E2f2*	*ngl*
sf1	*Src42A*	*brm*	*garz*	*Hr39*	*l(2)gd1*
kni	*tkv*	*bs*	*Pvf1*		

Table S2. Down-regulated genes in *mid* mutant among wing development-related genes.

Gene symbol	Forward primer	Reverse primer
β-tubulin	ACCTTCATCGGCAACTCCACTG	CGGTGAACTCCATCTCGTCCAT
mid	GACAGACAACAACAACGGCT	CAGTCGACCACATCAACGTC
wg	CAGTTAGTCCGAATGCAGCC	GTTCGGGTGATGGATCTTGC
arm	ACGAGGAGATGGAGGGAGAT	GATCAAACTGGGTGGAGGGA
hh	TCGTGTTTTGAGCATGACCG	AGCACCTGGTTCTTCTCCTC
dpp	AGAGATTCATCGCCGCCATA	TGCACTTCGTTGAACTGTCG
DE-Cad	AACACCTACGTTCTCACCGT	GTCACGATCGAATGTGTGGG

Table S3. Real-time PCR primers.
